# Iron Content Affects Lipogenic Gene Expression in the Muscle of Nelore Beef Cattle

**DOI:** 10.1371/journal.pone.0161160

**Published:** 2016-08-17

**Authors:** Wellison Jarles da Silva Diniz, Luiz Lehmann Coutinho, Polyana Cristine Tizioto, Aline Silva Mello Cesar, Caio Fernando Gromboni, Ana Rita Araújo Nogueira, Priscila Silva Neubern de Oliveira, Marcela Maria de Souza, Luciana Correia de Almeida Regitano

**Affiliations:** 1 Department of Genetic and Evolution, Federal University of São Carlos, São Carlos, Brazil; 2 Department of Animal Science, University of São Paulo/ESALQ, Piracicaba, Brazil; 3 Embrapa Pecuária Sudeste, São Carlos, Brazil; 4 Bahia Federal Institute of Education, Science and Technology, Ilhéus, Brazil; Oklahoma State University, UNITED STATES

## Abstract

Iron (Fe) is an essential mineral for metabolism and plays a central role in a range of biochemical processes. Therefore, this study aimed to identify differentially expressed (DE) genes and metabolic pathways in *Longissimus dorsi* (LD) muscle from cattle with divergent iron content, as well as to investigate the likely role of these DE genes in biological processes underlying beef quality parameters. Samples for RNA extraction for sequencing and iron, copper, manganese, and zinc determination were collected from LD muscles at slaughter. Eight Nelore steers, with extreme genomic estimated breeding values for iron content (Fe-GEBV), were selected from a reference population of 373 animals. From the 49 annotated DE genes (FDR<0.05) found between the two groups, 18 were up-regulated and 31 down-regulated for the animals in the low Fe-GEBV group. The functional enrichment analyses identified several biological processes, such as lipid transport and metabolism, and cell growth. Lipid metabolism was the main pathway observed in the analysis of metabolic and canonical signaling pathways for the genes identified as DE, including the genes *FASN*, *FABP4*, and *THRSP*, which are functional candidates for beef quality, suggesting reduced lipogenic activities with lower iron content. Our results indicate metabolic pathways that are partially influenced by iron, contributing to a better understanding of its participation in skeletal muscle physiology.

## Introduction

As an essential micronutrient, iron (Fe) plays a central role in several biochemical functions, by acting as an enzymatic cofactor and/or a structural protein component. Among these functions are energy metabolism, oxygen binding and transport, gene regulation, growth regulation, and cell differentiation [1]. Iron is also an essential component of several cell enzymes such as catalases, peroxidases, cytochromes, ribonucleotide reductase, and aconitase, which are crucial for physiological functions and have been implicated in a range of diseases due to alterations in iron metabolism [2].

Like other minerals, iron acts in the synthesis and metabolism of several hormones that, by complex interactions, are involved in metabolism, growth, and development [3]. Many of those hormones, such as the thyroid hormone (TH), growth hormone (GH), insulin-like growth factor (IGF), among others, have a recognized role in muscle development and functioning [3] and in determining the types of myofibers [4].

Recent studies have shown that muscle iron content can affect beef quality parameters [5,6]. Iron content was related to beef flavor, juiciness [5–7], red color intensity and lipid oxidation [8]. While the levels of cholesterol and polyunsaturated fatty acids were negatively correlated, monounsaturated fatty acid levels were positively associated with the iron content in the *Semitendinosus* muscle in Piedmontese, Simmental, and Angus cross animals [9].

Consumed worldwide, beef is an excellent source of iron, both regarding quantity and bioavailability [10], contributing up to 18% of daily needs [11]. However, according to the World Health Organization, anemia due to lack of iron is the world’s most common nutritional disorder, affecting around 30% of the population [12]. Therefore, besides the effects on beef quality, breeding for iron content can add nutritional value to beef, providing key micronutrients to human diet [7].

Regarding the genetic aspects of iron content variation in cattle populations, Casas et al. [13] showed an association between polymorphisms in calpastatin and micro-calpain genes with the iron amount in beef, where genotypes associated with tougher beef had higher iron concentrations. The chromosomes 7 and 12 of Nelore cattle [14] and chromosomes 1, 2, 7, 10, 15, and 28 of Angus [7] harbor Quantitative Trait Loci (*QTLs*) affecting iron content in the *Longissimus dorsi* (LD) muscle.

Despite those studies, the genetic variation underlying iron homeostasis and its role in pathways that affect meat quality remain poorly understood, notably in cattle. Advances in RNA sequencing (RNAseq) technologies have allowed a better understanding of the bovine transcriptome, including the identification of differentially expressed genes, isoforms, and new transcripts [15, 16]. Gene expression analysis has been proven to be a powerful tool for the identification of metabolic pathways related to iron content in model species [17–19].

Iron is an essential mineral for metabolism. However, there is still a lack of information on potentially functional genes and metabolic pathways related to the iron content in bovine tissues. The present study aimed to identify differentially expressed genes (DE) that influence iron content in Nelore steers *Longissimus dorsi* muscle (LD). Identifying these genes and biological functions may provide potential functional target genes, besides a better understanding of physiological consequences of iron content variation in beef cattle.

## Material and Methods

### Animals, samples, and phenotypes

All the experimental procedures involving animals were approved by the committee on animal use and welfare of Embrapa (Brazilian Agricultural Research Corporation).

Nelore steers (n = 373), components of a family of half-siblings, were produced by Embrapa. The production system and other experimental details have previously been described [14]. In brief, animals were raised in grazing systems in four farms with similar nutritional and sanitary management until 21 months of age when they were taken to two feedlots with similar diets. After about 90 days in the feedlot, the animals were slaughtered and samples of the LD muscle, between the 11th and 13th ribs, were collected. These samples were used to measure mineral content and for RNA extraction. Moreover, the intramuscular fat content (IMF), and backfat thickness deposition (BFT) were measured as previously published [20,21].

Samples were lyophilized, and aliquots of approximately 0.1 g were accurately weighed directly into the closed PFA (perfluoro alkoxy alkanes) microwave vessels and microwave assisted (Ethos 100, Milestone, Sorisole, Italy). The iron content was measured at 238.204 nm by inductively coupled plasma-optical emission spectrometry (ICP OES; Vista Pro-CCD ICP OES^®^, radial view, Varian, Mulgrave, Australia). Besides iron, 13 minerals, including copper (Cu), zinc (Zn) and manganese (Mn), were measured as previously described [14].

The variance components and the genomic estimated breeding value (GEBV) were obtained through a Bayesian approach implemented in the GenSel software [22], which has already been described by Tizioto et al. [14]. The statistical model used included the fixed effects of contemporary groups formed by birthplace (n = 4), feedlot location (n = 2) and breeding season (n = 2). Each animal’s age at slaughter time was included as a covariate [14]. The animals were classified according to GEBV for iron content, and eight divergent animals were selected: four with high GEBV and four with low GEBV, from now on called, respectively, HighFe (high GEBV for iron content) and LowFe (low GEBV for iron content).

The Student’s t-test was used to evaluate the mean differences between the groups for estimated GEBV values of Cu, Mn, and Zn content, IMF and BFT, available from previous studies [14,20,21]. The phenotypic correlation between the evaluated minerals using the whole population (n = 373) was estimated with the PROC CORR procedure of the SAS 9.0 (Statistical analysis software).

### RNA extraction, library preparation, and sequencing

The total RNA from eight selected animals was individually extracted from at least 100 mg of LD muscle sample, using TRIzol^®^ (Life Technologies, Carlsbad, CA), according to the manufacturer’s instructions, and analyzed in a Bioanalyzer 2100^®^ (Agilent, Santa Clara, CA, USA). To ensure appropriate RNA quality the minimum RNA integrity number (RIN) adopted was 8.

The RNA libraries for each sample were prepared using the TruSeq RNA Sample Preparation Kit (Illumina, San Diego, CA), using 2 μg of total RNA, according to the protocol of the TruSeq RNA Sample Preparation Kit v2 Guide (Illumina, San Diego, CA). The same Bioanalyzer 2100^®^ was used to estimate the average size of fragments from each library, and the quantitative PCR with KAPA Library Quantification kit (KAPA Biosystems, Foster City, CA, USA) was used to quantify the libraries. The cluster generation and sequencing were carried out in an Illumina HiSeq 2500^®^ (Illumina, San Diego, CA) to produce paired-end reads of 2x 100 bp. The libraries’ preparation and the sequencing were carried out at the ESALQ Genomics Center (Piracicaba, SP, Brazil).

### Quality control and read mapping

After removing the sequencing adaptors and low-complexity reads through the Seqclean software (http://sourceforge.net/projects/seqclean/files/), the reads were mapped to the bovine reference genome (*Bos taurus* UMD3.1, http://www.ensembl.org/Bos_taurus/Info/Index/) using the Tophat2 software (v2.0.11) [23]. The mapping was independently carried out for each sample, allowing up to two mismatches per read.

### Transcript assembly and abundance estimation

The assembly of the transcripts was performed with the Cufflinks2 software (v2.2.1) [24], for which a reference annotation file was provided. Moreover, Cufflinks2 estimated the abundance of the transcripts, whose expression level was determined by the normalized number of fragments per kilobase of exon per million fragments mapped (FPKM). After the transcripts were individually assembled for each sample, the Cuffmerge program (v2.0.2) generated a single notation file, uniting transcripts assembled parsimoniously [25].

### Identification of the differentially expressed genes

Differential expression analysis was carried out by the Cuffdiff2 software [25], which considered the abundance of each transcript, primary transcript or gene, compared to the expression levels between treatments, and tested the statistical significance of the observed changes by adopting a linear maximum likelihood model [25]. The correction for multiple tests was performed using the Benjamini-Hochberg method (q-value ≤ 0.05) [26], implemented in the Cuffdiff2 software.

The FPKM values were used to determine the fold change (log_2_fold change). For DE genes, the sign of the log_2_ (fold change) was adopted as a criterion for gene classification into up- and down-regulated (False Discovery Rate ≤ 0.05).

The CummeRbund package, implemented in the R software, was adopted for data visualization, manipulation, and exploration [27].

### Functional gene classification, canonical pathway analysis, and gene networks

The functional categorization based on the terms of the Gene Ontology (GO) was carried out together for the up- and down-regulated genes. Moreover, the terms of the GO-slim ontology (high-level GO terms) were used to select the most important biological processes [28] using the WEB-based Gene seT AnaLysis Toolkit (WebGestalt) software [29]. The top ten categories for each GO term based on the p-value adjusted for multiple tests (adjP <0.05) were considered as enriched.

We also used the Functional Annotation Cluster, a tool of the Database for Annotation Visualization and Integrated Discovery (DAVID) software (v.6.7) [30], to build biological modules based on groups of related functional terms for both categories of DE genes [31]. Clusters with EASE score < 0.1, which is calculated from a modification of Fisher’s exact test [30], were considered significant.

An additional approach to identify overrepresented metabolic pathways and gene networks was conducted using the QIAGEN’s Ingenuity^®^ Pathways Analysis software (IPA^®^, QIAGEN, Redwood City; www.qiagen.com/ingenuity). The IPA allows the exploration of the mechanisms, pathways and functions in which the DE genes participate, from the established relations among the genes, RNAs and proteins included in the Ingenuity Pathways Knowledge Base (IPKB) (QIAGEN, Redwood City; www.qiagen.com/ingenuity). Within the IPKB, the information contained in the scientific literature is manually curated and modeled to help in the understanding and interpretation of the data’s biological meaning (http://www.ingenuity.com/products/ipa). The genes were converted to their corresponding human homologs in the IPA database to perform a core analysis function. Significance was given to metabolic pathways and biological processes with p < 0.05, calculated by the right-tailed Fisher exact test, which compares the number of DE genes with the total number of these genes’ occurrences in the IPKB.

## Results

### Phenotypic group, mapping, and transcript assembly

Animals with different iron content phenotypes were selected based on GEBVs estimated in a previous genome-wide association study (GWAS) with the same experimental population. The GWAS analysis, as well as the descriptive statistics for all the population (n = 373), has been previously reported [14]. The genetic and residual variances, as well as the heritability for iron content obtained from this population (n = 373), were 0.15, 0.31 and 0.32, respectively [14]. There was no difference between the groups regarding the mineral content (Cu, Mn, and Zn) as well as the IMF and BFT traits (P > 0.05) (S1 Table).

The GEBVs obtained from the 373 animals were ranked, and four animals with high and other four animals with low GEBV for iron content (HighFe and LowFe groups, respectively) were selected for sequencing. A total of 150.49 million reads were produced by the Illumina HiSeq 2500^®^ System (Illumina, Inc., San Diego, CA). The average number of raw reads per sample was 18.8 million. The average number of mapped reads estimated by Tophat2 was 9.13 million, which corresponds to 90.3% of the total. The summary of the phenotypic iron content data, the GEBVs, and the number and percentage of mapped reads are presented in Table 1.

**Table 1:** Iron content in the Nelore steers *Longissimus dorsi* muscle, iron content genomic estimated breeding value (Fe-GEBV), number of reads after cleaning, number and percentage of mapped reads.

Animal	Category	Fe Content^a^	Fe-GEBV	Reads^b^	Mapped reads^c^	%^d^
Low1	LowFe^e^	9.75	-1.436	6.66	6.02	90.4
Low2	LowFe	18.30	-1.261	6.18	5.59	90.4
Low3	LowFe	1.41	-1.006	14.27	12.84	89.9
Low4	LowFe	0.90	-0.459	13.85	12.53	90.5
**Mean**		**7.59**	**-1.041**	**10.24**	**9.25**	**90.3**
High1	HighFe^f^	89.60	0.167	11.95	10.77	90.1
High2	HighFe	51.57	0.162	4.42	3.99	90.3
High3	HighFe	56.44	0.166	18.29	16.51	90.3
High4	HighFe	37.19	0.169	5.28	4.82	91.3
**Mean**		**58.70**	**0.166**	**9.99**	**9.02**	**90.5**

^a^mg/kg

^b^millions of reads after cleaning

^c^milions of reads mapped

^d^percentage of mapped reads

^e^LowFe–low iron content group

^f^HighFe–high iron content group.

In all, 606,378 transcripts were identified and classified by Cuffmerge (S1 Fig), with a particular notice to a large number of potential new isoforms (59% of the transcripts) and complete correspondence in an intron (39.6%). Alternative splicing events are common in eukaryotes, indicating the functional complexity of higher organisms such as cattle [32].

For the evaluated conditions, 25,208 genes were identified, 2,802 of which coded for predicted or not yet characterized proteins, and 4,857 were new genes not yet annotated, according to Cufflinks2 [24]. The sequencing quality and the transcript distribution among the studied groups are presented as a box plot of log_10_ of the FPKM values (S2 Fig). The data show a negative asymmetric distribution with similar median and quartile values between the HighFe and LowFe groups.

### Identification of differentially expressed genes

The variation observed in gene expression patterns among animals allowed the identification of 63 DE genes (q < 0.05), 49 of which were annotated and used in the functional enrichment analyses and metabolic pathway identification.

For the DE genes, 18 were ranked as up-regulated (Table 2) and 31 as down-regulated (Table 3) in the LowFe animals. Differences, similarities, and variability between the groups are represented in the hierarchical clustering carried out from the DE genes and the individual values represented in the FPKM log_10_ heat map (S3 Fig). Principal component analysis (PCA; S4 Fig) and multi-dimensional scaling (MDS; S5 Fig) confirmed the replicate separation, according to the conditions tested in this study. Also, PCA and MDS analyses showed that the approach used captured the genetic variation resulting from the phenotypic differences.

**Table 2:** Significantly up-regulated genes identified in the Nelore steers *Longissimus dorsi* muscle with low genomic estimated breeding value compared to animals with high genomic breeding value for iron content.

Ensembl Gene ID	Gene Symbol	Gene name	HighFe^a^ (FPKM)	LowFe^b^ (FPKM)	log_2_FC^c^	q-value
ENSBTAG00000000569	*HES1*	Hairy and enhancer of split 1, (Drosophila)	29.718	49.604	0.739	0.0125
ENSBTAG00000038464	*PLIN5*	Perilipin 5	13.355	22.802	0.772	0.0125
ENSBTAG00000006013	*NT5DC3*	5'-nucleotidase domain containing 3	6.244	10.661	0.772	0.0125
ENSBTAG00000008953	*TAP1*	Transporter 1, ATP-binding cassette, sub-family B (MDR/TAP)	5.335	9.259	0.795	0.0487
ENSBTAG00000036363	*MIR133A-1*	-	20.789	36.738	0.821	0.0125
ENSBTAG00000021481	*CA14*	Carbonic anhydrase XIV	17.449	30.935	0.826	0.0125
ENSBTAG00000012335	*UBA7*	Ubiquitin-like modifier activating enzyme 7	3.476	6.293	0.856	0.0487
ENSBTAG00000017461	*SLC16A3*	Solute carrier family 16, member 3 (monocarboxylic acid transporter 4)	18.307	37.000	1.015	0.0125
ENSBTAG00000014628	*OAS1*	2'-5'-oligoadenylate synthetase 1, 40/46kda	2.495	5.990	1.263	0.0125
ENSBTAG00000014538	*HPCAL4*	Hippocalcin like 4	1.249	3.079	1.302	0.0125
ENSBTAG00000031209	*SLC22A4*	Solute carrier family 22 (organic cation/ergothioneine transporter), member 4	6.663	16.550	1.313	0.0125
ENSBTAG00000014818	*MYLK3*	Myosin light chain kinase 3	1.713	4.560	1.413	0.0125
ENSBTAG00000005078	*UCHL1*	Ubiquitin carboxyl-terminal esterase L1 (ubiquitin thiolesterase)	3.078	8.542	1.472	0.0312
ENSBTAG00000030913	*MX1*	Myxovirus (influenza virus) resistance 1, interferon-inducible protein p78 (mouse)	2.026	5.824	1.523	0.0125
ENSBTAG00000004860	*SLC27A6*	Solute carrier family 27 (fatty acid transporter), member 6	2.676	8.000	1.580	0.0125
ENSBTAG00000019588	*BLA-DQB*	MHC class II antigen	5.327	17.210	1.692	0,0125
ENSBTAG00000013598	*RSPO2*	R-spondin 2	1.345	5.381	2.001	0.0125
UNCHARACTERIZED	*LOC100848684*	-	0.994	6.333	2.672	0.0125

^a^HighFe–high iron content group

^b^LowFe–low iron content group

^c^log2 FC–log2 fold change between HighFe and LowFe.

**Table 3:** Significantly down-regulated genes identified in the Nelore steers *Longissimus dorsi* muscle with low genomic breeding value compared to animals with high genomic breeding value for iron content.

Ensembl Gene ID	Gene Symbol	Gene name	HighFe^a^ (FPKM)	LowFe^b^ (FPKM)	log_2_FC^c^	qvalue
ENSBTAG00000009876	*C4BPA*	Complement component 4 binding protein, alpha	2.067	0.197	-3.393	0.0402
ENSBTAG00000017280	*C3*	Complement component 3	3.679	0.506	-2.862	0.0125
ENSBTAG00000038067	*MT1A*	Metallothionein 1A	32.842	4.991	-2.718	0.0125
ENSBTAG00000011976	*CYP4B1*	Cytochrome P450, family 4, subfamily B, polypeptide 1	13.993	3.256	-2.104	0.0311
ENSBTAG00000011666	*THRSP*	Thyroid hormone responsive	12.927	3.392	-1.930	0.0231
ENSBTAG00000014340	*KERA*	Keratocan	5.333	1.520	-1.811	0.0125
ENSBTAG00000018223	*CHI3L1*	Chitinase 3-like 1 (cartilage glycoprotein-39)	5.485	1.610	-1.769	0.0125
UNCHARACTERIZED	*LOC100848726*	-	101.140	30.394	-1.734	0.0125
ENSBTAG00000019587	*PI15*	Peptidase inhibitor 15	2.098	0.732	-1.518	0.0311
ENSBTAG00000000575	*TNC*	Tenascin C	2.798	1.059	-1.401	0.0125
ENSBTAG00000003120	*ZNF385B*	Zinc finger protein 385B	3.797	1.616	-1.233	0.0231
ENSBTAG00000020035	*RCAN1*	Regulator of calcineurin 1	125.998	54.342	-1.213	0.0125
ENSBTAG00000001257	*AGTPBP1*	ATP/GTP binding protein 1	20.313	8.810	-1.205	0.0125
ENSBTAG00000039035	*HSPA6*	Heat shock 70kda protein 6 (HSP70B')	9.216	4.019	-1.197	0.0125
ENSBTAG00000014614	*ACTG2*	Actin, gamma 2, smooth muscle, enteric	18.307	8.019	-1.191	0.0125
ENSBTAG00000014906	*VCAN*	Versican	7.353	3.289	-1.161	0.0125
UNCHARACTERIZED	*LOC100848883*	-	6.006	2.726	-1.139	0.0231
UNCHARACTERIZED	*LOC100848012*	-	6.358	2.977	-1.095	0.0311
ENSBTAG00000010285	*MMRN1*	Multimerin 1	5.529	2.732	-1.017	0.0125
ENSBTAG00000006729	*ARID5B*	AT rich interactive domain 5B (MRF1-like)	21.826	10.826	-1.012	0.0125
ENSBTAG00000015988	*MYH11*	Myosin, heavy chain 11, smooth muscle	16.100	8.164	-0.980	0.0125
ENSBTAG00000011207	*CNN1*	Calponin 1, basic, smooth muscle	16.474	8.370	-0.977	0.0125
ENSBTAG00000032774	*C28H10orf116*	Chromosome 28 open reading frame	77.715	40.357	-0.945	0.0402
ENSBTAG00000015434	*DSTN*	Destrin (actin depolymerizing factor)	59.435	31.252	-0.927	0.0125
ENSBTAG00000002215	*GFPT2*	Glutamine-fructose-6-phosphate transaminase 2	7.603	4.063	-0.904	0.0125
ENSBTAG00000019107	*GAS7*	Growth arrest-specific 7	3.790	2.040	-0.894	0.0311
ENSBTAG00000002178	*PPL*	Periplakin	3.093	1.666	-0.892	0.0231
ENSBTAG00000015980	*FASN*	Fatty acid synthase	11.512	6.475	-0.830	0.0125
ENSBTAG00000037526	*FABP4*	Fatty acid binding protein 4, adipocyte	86.012	49.279	-0.804	0.0125
ENSBTAG00000002080	*NOV*	Nephroblastoma overexpressed	18.160	10.629	-0.773	0.0125
ENSBTAG00000020056	*COL12A1*	Collagen, type XII, alpha 1	7.443	4.596	-0.696	0.0311

^a^HighFe–high iron content group

^b^LowFe–low iron content group

^c^log2 FC–log2 fold change between HighFe and LowFe.

Lipogenic genes, such as cytochrome P450, family 4, subfamily B, polypeptide 1 (*CYP4B1*), fatty acid binding protein 4, adipocyte (*FABP4*), fatty acid synthase (*FASN*), and thyroid hormone responsive (*THRSP*) were down-regulated in animals with LowFe phenotype. Besides those, the component complement 3 (*C3*) gene was also observed to be down-regulated. On the other hand, the perilipin 5 (*PLIN5*) and solute carrier family 27 (fatty acid transporter), member 6 (*SLC27A6*) genes were up-regulated. Genes related to cell growth and proliferation, such as versican (*VCAN*), tenascin C (*TNC*), and regulator of calcineurin 1 (*RCAN1*), were shown as being down-regulated in the LowFe group. In this same group, miR-133a was identified to be up-regulated.

### Functional enrichment and metabolic pathways

**Fig 1:**
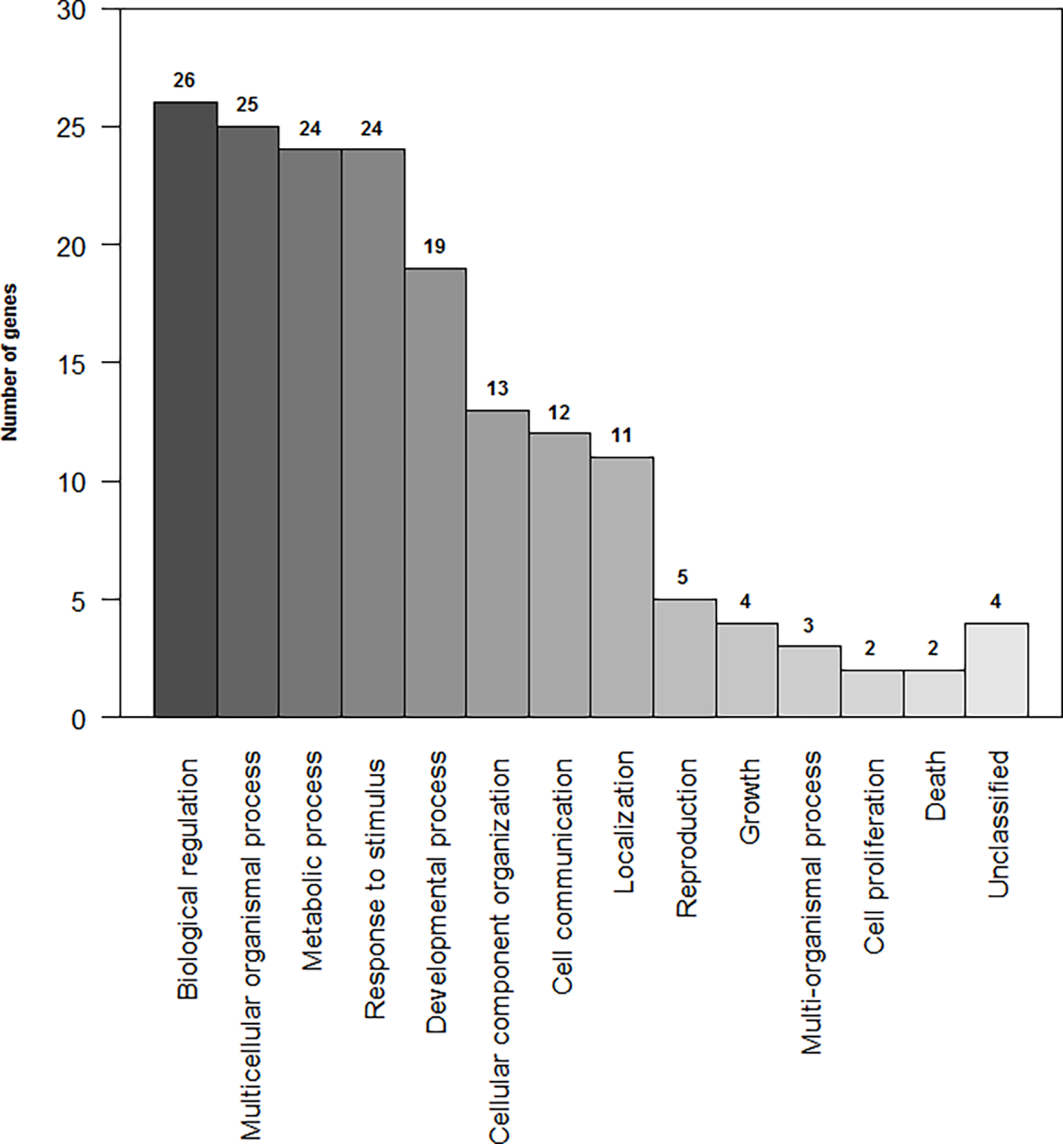
Ranking of the biological processes based on the terms of Gene Ontology (GO-Slim) for the differentially expressed genes identified in Nelore steers with high and low genomic breeding value for iron content in the *Longissimus dorsi* muscle. The bar represents each category of the biological process. The number of genes per category is indicated above the bar.

To better understand the biological roles of the DE genes, functional enrichment analyses were conducted together for all 49 identified genes. Among the DE genes, 45 of them were categorized into 13 functional groups (Fig 1). The most represented terms for biological processes by the GO-slim were biological regulation, multicellular organism processes, and metabolic processes. The functional groups identified from the DE genes by the WebGestalt program (adjP < 0.05) were associated with the metabolism of lipids, triglycerides, and acylglycerol. Besides those, other indicated groups were striated muscle cell development and actomyosin structure organization (Table 4).

**Table 4:** Biological processes significantly enriched by WebGestalt for the differentially expressed genes in the *Longissimus dorsi* muscle, comparing high and low genomic breeding value groups for iron content in Nelore steers.

GO ID	Term	Genes*	C^a^	E^b^	R^c^	rawP^d^	adjP^e^
0006638	Neutral lipid metabolic process	***C3***, ***FABP4***, ***FASN***, *SLC22A4*, ***THRSP***	110	0.29	17.05	1.03e-05	0.0017
0006639	Acylglycerol metabolic process	***C3***, ***FABP4***, ***FASN***, *SLC22A4*, ***THRSP***	109	0.29	17.21	9.85e-06	0.0017
0006641	Triglyceride metabolic process	***C3***, ***FABP4***, ***FASN***, *SLC22A4*, ***THRSP***	104	0.28	18.04	7.83e-06	0.0017
0055001	Muscle cell development	*HES1*, ***MYH11***, *MYLK3*, ***RCAN1***, ***TNC***	145	0.39	12.94	3.92e-05	0.0049
0031032	Actomyosin structure organization	***CNN1***, ***MYH11***, *MYLK3*	47	0.13	23.94	0.0003	0.0252
0055002	Striated muscle cell development	***MYH11***, *MYLK3*, ***RCAN1***, ***TNC***	121	0.32	12.40	0.0003	0.0252
0019432	Triglyceride biosynthetic process	***C3***, ***FASN***, ***THRSP***	56	0.15	20.10	0.0004	0.0280
0046460	Neutral lipid biosynthetic process	***C3***, ***FASN***, ***THRSP***	58	0.15	19.40	0.0005	0.0280
0046463	Acylglycerol biosynthetic process	***C3***, ***FASN***, ***THRSP***	58	0.15	19.40	0.0005	0.0280
0044707	Single-multicellular organism process	***ACTG2***, ***AGTPBP1***, ***ARID5B***, ***C3***, ***CHI3L1***, ***CNN1***, ***COL12A1***, ***CYP4B1***, ***FABP4***, ***GAS7***, *HES1*,*HPCAL4*, ***KERA***, ***MMRN1***, ***MYH11***,*MYLK3*, ***PPL***, ***RCAN1***, *RSPO2*,*SLC16A3*, *SLC22A4*, *TAP1*, ***TNC***, *UBA7*, ***VCAN***	5612	14.96	1.67	0.0010	0.0292

*Highlighted in bold, down-regulated genes in the LowFe group animals

^**a**^C: number of reference genes in the category

^**b**^**E**:expected number in the category

^**c**^**R**: Ratio of enrichment

^**d**^**rawP**: p-value from hypergeometric test

^**e**^**adjP**: p-value adjusted by the multiple test adjustment

We used the David functional annotation cluster tool to build biological modules by clusters of functional terms related to the differentially expressed genes [30,31]. Seven enriched functional groups were significant (EASE score < 0.1) (S1 Table). The genes were organized in seven clusters comprehending functions related to development and differentiation of muscle cells, extracellular matrix, cell morphogenesis and proliferation, nucleotide linkages, actin cytoskeleton, and immune response.

**Fig 2:**
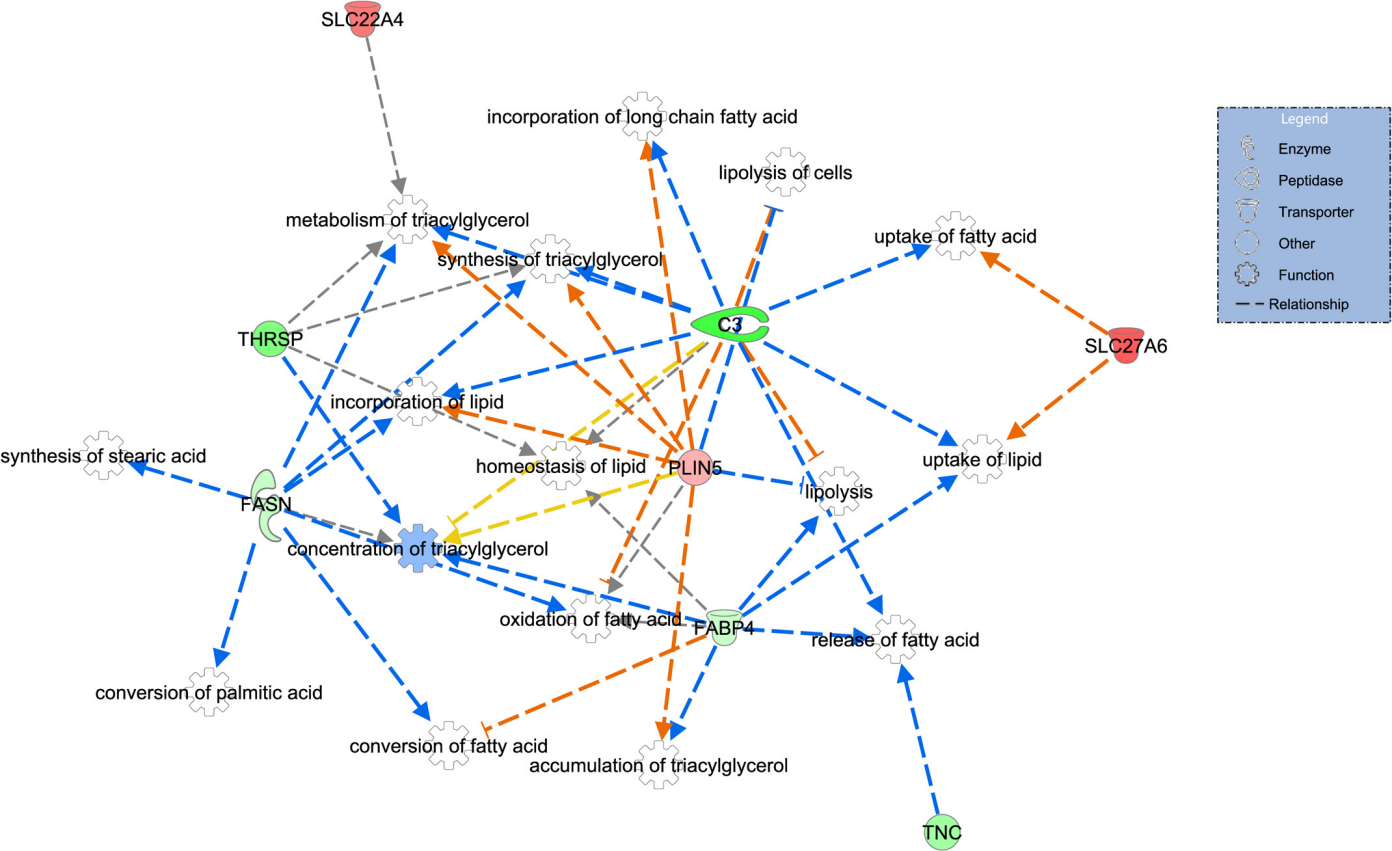
Lipid metabolism related network of differentially expressed genes between high and low iron content in the Nelore steers *Longissimus dorsi* muscle. Genes in green and red are down- and up-regulated in the LowFe group, respectively. Color intensity refers to fold change estimates. Dotted arrows represent indirect interactions, and orange, blue, gray, and yellow colors denote activation, inhibition, unpredicted effect, and inconsistent effect, respectively. Reprinted from [QIAGEN’s Ingenuity^®^ Pathways Analysis] under a CC BY license, with permission from QIAGEN Silicon Valley, original copyright [200–2015 QIAGEN].

**Fig 3:**
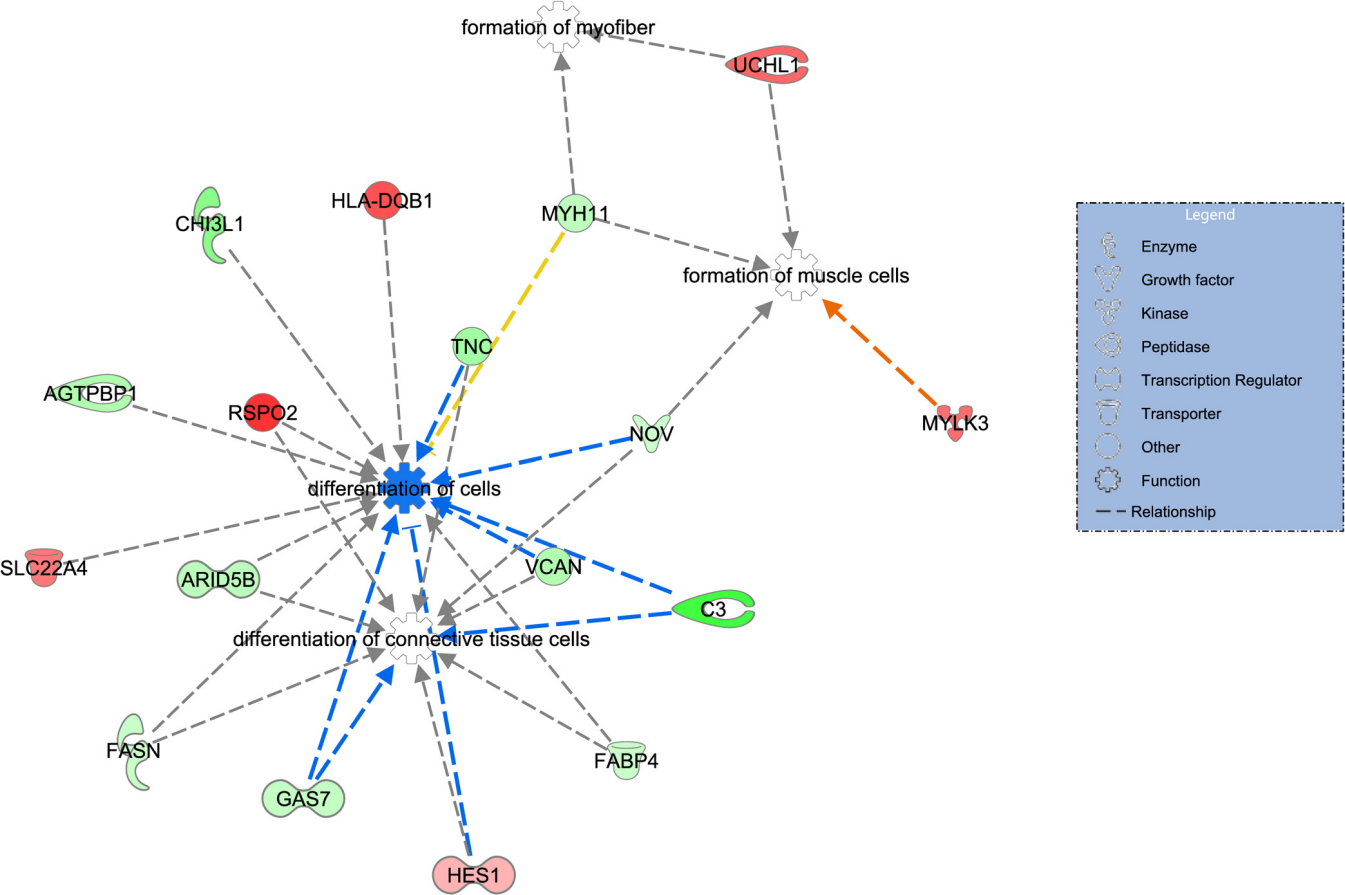
Cell development and a differentiation-related network of differentially expressed genes between high and low iron content in the Nelore steers *Longissimus dorsi* muscle. Genes presented in green and red are down- and up-regulated in the LowFe group, respectively. Color intensity refers to fold change estimates. Dotted arrows represent indirect interactions, and colors orange, blue, gray, and yellow denote activation, inhibition, unpredicted effect, and inconsistent effect, respectively. Reprinted from [QIAGEN’s Ingenuity^®^ Pathways Analysis] under a CC BY license, with permission from QIAGEN Silicon Valley, original copyright [200–2015 QIAGEN].

To identify metabolic pathways not yet indicated by other programs, a third approach was adopted, the core analysis function, included in the IPA program. From 49 DE genes, five (*LOC100848883*, *LOC100848012*, *LOC100848726*, *LOC100848684*, *MIR133A-1*) did not present human homologs in the IPA database and were not taken into consideration in the analysis. Of the DE genes analyzed by IPA, 16 were up-regulated and 29 down-regulated in the LowFe group. The main biological functions evidenced by IPA (P value < 0.05) were lipid metabolism (Fig 2) and cell development and growth (Fig 3). Besides those, evidence showed processes related to the biochemistry of small molecules and the development and function of the hematological system, and cell-cell interaction and signaling (Table 5), among other aspects, which had not been observed in the previous analyses.

**Table 5:** Five main significant biological functions identified by IPA for the differentially expressed genes in the *Longissimus dorsi* muscle, comparing high and low genomic breeding value groups for iron content in Nelore steers.

Biological function	Category	Genes^a^	P value range
**Molecular and cellular functions**	Lipid metabolism	***AGTPBP1***, ***C3***, ***CYP4B1***, ***FABP4***, ***FASN***, *MX1*, *PLIN5*, *SLC27A6*, *SLC22A4*, ***THRSP***, ***TNC***	2.30E-06 2.62E-02
	Small molecule biochemistry	***C3***, *CA14*, ***CYP4B1***, ***FABP4***, ***FASN***, *MX1*, *PLIN5*, *SLC27A6*, *SLC22A4*, ***THRSP***, ***TNC***, ***VCAN***, ***AGTPB1***, *GFPT2*	2.30E-06 2.62E-02
	Cellular development	***ARID5B***, ***AGTPB1***, *BLA-DQB*, ***C3*, *CHI3L1***, ***FABP4*, *FASN***, ***GAS7***, *HES1*, ***MYH11***, *MYLK3*, ***NOV***, ***PPL***, ***RCAN1***, *RSPO2*, *SLC22A4*, ***TNC*,** *UCHL1*, ***VCAN***	2.60E-06 2.31E-02
	Cellular growth and proliferation	***ARID5B***, *BLA-DQB*, ***C3***, ***CHI3L1***, ***CNN1***, ***FABP4***, ***FASN***, ***GAS7***, *HES1*, ***MT1A***, *MX1*, ***MYH11***, ***NOV***, ***PPL***, ***RCAN1***, *SLC22A4*, *TAP1*, ***TNC***, *UCHL1*, ***VCAN***	1.43E-05 2.40E-02
	Cell-to-cell signaling and interaction	*BLA-DQB*, ***CHI3L1***, ***C3***, ***C4BP4***, ***FASN***, *HES1*, *MYLK3*, ***NOV***, ***RCAN1***, *TAP1*, ***TNC***, *UCHL1*, ***VCAN***	1.32E-04 2.83E-02
**Physiological system development and function**	Tissue development	***AGTPBP1***, ***ARID5B***, *BLA-DQB*, ***C3***, ***C4BPA***, ***COL12A1***, ***DSTN***, ***FABP4***, ***FASN***, ***GAS7***, *HES1*, ***KERA***, ***MYH11***, *MYLK3*, ***NOV***, *RSPO2*, ***TNC***, ***VCAN***, ***RCAN1***, *UCHL1*	2.60E-06 2.83E-02
	Hematological system development and function	***ARID5B***, *BLA-DQB*, ***CHI3L1***, ***C3***, ***C4BPA***, ***FABP4***, *HES1*, ***KERA***, ***MYH11***, ***RCAN1***, *TAP1*, ***TNC***, ***VCAN***,	1.32E-04 2.63E-02
	Immune cell trafficking	***CHIL3L1***, ***C3***, ***FABP4***, ***MYH11***, *MYLK3*, ***RCAN1***, ***TNC***, ***VCAN***	1.32E-04 2.83E-02
	Organismal development	***AGTPBP1***, ***ARID5B***, *BLA-DQB*, ***C3***, *CA14*, ***COL12A1***, ***CNN1***, ***FABP4***, *HES1*, ***KERA***, ***MYH11***, *MYLK3*, ***NOV***, *PLIN5*, *SLC22A4*, ***RCAN1***, *RSPO2*, *UCHL1*, *TAP1*, ***TNC***	2.51E-04 2.83E-02
	Skeletal and muscular system development and function	***ARID5B***, ***C3***, ***CNN1***, ***COL12A1***, ***FASN***, ***GAS7***, ***MYH11***, *MYLK3*, ***NOV***, *PLIN5*, ***RCAN1***, *RSPO2*, ***TNC***, *UCHL1*, ***VCAN***	4.31E-04 2.83E-02

^a^Highlighted in bold, down-regulated genes in the LowFe group animals.

IPA identified several canonical pathways, of which six stood out. Among the canonical signaling pathways, the main ones were interferon signaling (*P**<* 6.82E-05, *OAS1*, *MX1* and *TAP1*), thyroid receptor activation (TR/RXR) (*P* *<* 8.75E-04, *SLC16A3*, *FASN* and *THRSP*), and complement system (*P* *<* 2.41E-03, *C3*, and *C4BPA*). The genes identified in the *OAS1*, *MX1*, *TAP1*, and *SLC16A3* canonical signaling pathways were up-regulated, while *C3*, *C4BPA*, *FASN*, and *THRSP* were down-regulated in the LowFe group. Likewise, the main overrepresented canonical metabolic pathways were stearate biosynthesis in animals (P < 2.71E-03, *FASN*, and *SLC27A6*), palmitate biosynthesis (P < 4.41E-03, *FASN*), and fatty acids biosynthesis (P < 4.41E-03, *FASN*). The *FASN* and *SLC27A6* genes were respectively down- and up-regulated in the LowFe group.

## Discussion

Market demands for healthier and more nutritious foods are growing. To meet these requirements, one must identify the nutritional components with negative and positive effects on health and then develop and apply tools to reduce or improve them, respectively [11]. Recent studies have demonstrated the opportunities to apply genomic tools to the improvement of difficult-to-measure complex traits, such as the selection for beef mineral content [7,9,14,33].

The RNA sequencing transcriptome analysis of the LD muscle in bovine representatives of extreme GEBV for iron content was adopted as a way to identify the genetic mechanisms underlying iron homeostasis regulation in the muscle. It can also be applied to infer the involvement of specific biological pathways resulting from the differences in the individual genetic composition [16]. To our knowledge, this is the first study seeking to identify differentially expressed genes and establish interactions that shed light on the biological mechanisms that either regulate or are regulated by the iron content in the LD muscle. We also evaluate the iron participation in biological pathways that affect beef quality.

Previous studies have indicated a genetic component that influences the amount of iron in the muscle of Nelore animals [14]. Similarly, high heritability for iron content in LD muscle was identified in Angus animals, so that this mineral was suggested as a candidate for selection [7]. Moreover, iron is a structural component or enzymatic cofactor of several enzymes [1] that play roles in muscle metabolism, leading us to believe that its availability would affect several physiological functions and, therefore, beef quality.

The simultaneous observation of the heat map and the principal component analysis presented respectively in S3 and S4 Figs demonstrate there was enough variation in the gene expression to separate the animals into two divergent phenotypes. Furthermore, the clustering performed using multi-dimensional scaling (MDS) approach supported the previous results (S5 Fig).

For the identified functional categories, we now discuss some genes considering aspects related to their physiological roles in the muscle, their influence on beef quality, and how the iron content might act on the identified metabolic pathway.

### Lipid metabolism

Differential expression analysis disclosed significant changes in the expression of lipid metabolism-related genes (Fig 2, Tables 4 and 5), which are key components in canonical metabolic pathways, related to fatty acid, stearate and palmitate biosynthesis, as identified in the present work.

The *THRSP*, *FASN*, *FABP4*, and *C3* genes, down-regulated in LowFe group, may be indirectly inhibiting functions related to lipid metabolism, such as metabolism of triacylglycerol, synthesis of stearic acid, oxidation and release of fatty acids, in addition to other functions as predicted by the IPA software. To our knowledge, there are no similar studies in cattle to evaluate global gene expression differences in animals genetically divergent for muscle iron content. Therefore, we discuss these results in light of the information available in the literature for other species from experiments in which nutritional conditions induced the variation of concentration of iron in the body.

The *THRSP* gene encodes a nuclear protein that acts as a lipogenic transcription factor, regulating genes such as *FASN* and *FABP4* [34,35], which are involved in the *de novo* synthesis of fatty acids [36–38], free fatty acid uptake, transport, and metabolism, respectively [39]. These genes are responsive to the levels of the thyroid hormone (TH), whose synthesis is impaired with the lesser availability of iron due to a reduction in thyroid peroxidase activity, which is heme-dependent [40,41]. It is probable, therefore, that decrease in the levels of *THRSP*, *FASN*, and *FABP4* expression in the LowFe animals is an adaptation to a smaller quantity of iron, since iron deficiency, with or without anemia, harms thyroid metabolism [40].

Supporting our finding of down-regulation of *C3* gene in the LowFe group, Li and coworkers [42] found the *C3* mRNA levels increased by iron treatment in human retinal pigment epithelial cells. Furthermore, *C3* has been implicated in lipid metabolism acting on free fatty acids transport and triacylglycerol synthesis (reviewed in [43]), functions that were predicted inhibited in the IPA analysis. The activation of lipolysis predicted in our data is in agreement with *C3* down-regulation [43,44].

In contrast, the induction of *PLIN5* and *SLC27A6* gene expression leads to activation of the lipogenic processes, such as synthesis and accumulation of triacylglycerol, and uptake of fatty acid. The *PLIN5* gene, more expressed in oxidative tissues, negatively regulates fat droplet hydrolysis [45] leading to lipid accumulation. *PLIN5’s* role in lipolysis regulation has been confirmed in studies with knockout mice and obese humans, in which is observed an increase in the lipolytic activity with a consequent reduction in the adipose tissue [45,46].

Studies with rats have revealed that iron deficiency can affect the function of many enzymes involved in lipid metabolism [17–19,47,48]. Davis and colleagues [47] showed a reduction in the expression of genes involved in *β*-oxidation and increased expression of lipogenic genes in both muscle and liver. Otherwise, Kamei et al. [19] demonstrated that hepatic genes, including *FASN*, that are related to fatty acids synthesis were down-regulated in iron-deficient rats. This latter finding supports our observation that lipogenic genes are down-regulated in LowFe content group. However, caution should be taken in this comparison since these results are from different tissues and species, that may not share the same iron requirements.

Among the canonical pathways identified in the IPA analyses, the activation pathways of the thyroid receptor (TR/RXR), stearate biosynthesis, palmitate, and fatty acids have functions that highlight both their participation in important metabolic functions [3] with effects on meat quality parameters [49] and the iron influence on those pathways [50].

The expression of lipogenic genes is affected by many factors such as the interaction between iron and copper (Cu) [51]. Although it is well established in non-ruminants, few studies aiming to assess the molecular mechanisms behind this relationship were performed in ruminants [52,53]. In cattle, Lee and colleagues [53] evaluated the effect of Cu on the expression of lipogenic genes. Although they observed the trend of lower backfat deposition in animals supplemented with Cu, they did not observe a significant influence of Cu on the expression of genes related to lipid metabolism in adipose tissue. To ensure that the differences in expression of lipogenic genes seen in this study can truly be attributed to iron content, we investigate the mean of Cu content, and we did not verify significant differences in Cu average between the low and high Fe groups (P > 0.05). Furthermore, we identified low phenotypic correlation between the Fe and Cu content (r = 0.132, P < 0.01). Besides Cu, we also investigated the possible influence of Mn and Zn, and there were no significant differences between low and high Fe content group (P > 0.05) (S1 Table).

These animals were evaluated for the IMF [20] and BFT [21]. Although we observed the change in the expression of lipogenic genes, the animals used in this study did not present significant difference for IMF and BFT (S1 Table). This result should be taken with caution due to the limited number of samples in each group. However, a lack of functional consequences may be in part attributed to the observed antagonistic effects of iron content on gene expression, such as the ones depicted in Fig 2, in which accumulation of triacylglycerol was predicted activated by the up-regulation of *PLIN5* and predicted repressed by the down-regulation of *FABP4* gene. Similar antagonistic effects were predicted for the regulation of incorporation of long chain fatty acid by *PLIN5* and *C3* genes, as well as for the synthesis of triacylglycerol regarding *FASN*, *PLIN5*, and *C3* genes. Since the experimental animals were under the normal physiological condition, these findings might represent the adjustments of metabolism aiming homeostasis under normal variable iron concentration.

As the lipid and mineral metabolisms have a complex regulatory mechanism, and we have a limited number of replicates in a one point collection, our results should be treated with caution. Nevertheless, we demonstrated that the observed DE genes are likely associated with differences in iron content between the groups. However, further studies are needed to determine the complete role of these minerals in gene expression and lipid metabolism.

### Cell development and growth

Biological processes related to cell development and structure were evidenced through enrichment analyses (Tables 4 and 5 and S2 Table). Besides those, clusters related to the extracellular matrix (ECM) (*COL12A1*, *KERA*, *TNC*, *VCAN*), actin cytoskeleton organization (*ACTG2*, *DSTN*, *GAS7*, *MYH11*, *PPL*), and cell morphogenesis (*HES1*, *GAS7*, *RCAN1*) were identified (S2 Table). As already discussed, iron is required for the functioning of these biological processes [1]. Furthermore, iron is essential for collagen synthesis and can act in ECM remodeling as a result of the increase in metalloproteinase activity [54].

IPA predicted the *TNC* and *VCAN* genes shown in Fig 3 as inhibiting cellular differentiation processes and connective tissue cells. In addition to these functions, these genes are predicted to participate in cell migration and proliferation [55,56], and interact with each other and also with collagen [57]. Furthermore, the *MT1A* gene was observed to be down-regulated in the LowFe group, which is in agreement with the opposite roles Metallothionein-1A and iron have in oxidative stress, the former acting in the prevention [58] and the latter in the induction [59] of this process. All these genes contribute to the assembly of extracellular matrix (ECM), with the central role of regulating and differentiating muscle and adipose cells [60].

Iron has been implicated in the modulation of biogenesis and expression of miRNAs and these act in the regulation of genes involved in iron homeostasis, resulting a bidirectional circuit [61,62]. The miR-133a identified as DE and up-regulated in LowFe group is involved in muscle-specific development, and muscle remodeling [63,64]. In this context, we observed that the *TNC*, a gene encoding EMC protein related to cell migration, adhesion, and proliferation, is a target of miR-133a [65], and this could be a plausible explanation of the observed down-regulation of *TNC* in animals with LowFe. Furthermore, the 3'-UTR sequence of the ferritin light chain (*FTL*) gene is homologous to the sequence of miR-133a [66]. Although we did not identify the *FTL* gene as DE, other mechanisms driven by miRs, such as translation repression, could result in reduced ferritin production, which would be in agreement with lower iron storage in tissues were miR-133a is up-regulated.

Categorized in clusters with functions of development and differentiation in muscle cells, *RCAN1* gene negatively modulates calcineurin and, as a result, calcineurin-dependent pathways [67]. Calcineurin plays roles related to regeneration and regulation of skeletal muscle hypertrophy [68]. The *RCAN1* gene had its expression regulated by the TH [69] and was down-regulated in the LowFe animals. Just as happens with the lipogenic genes, previously discussed, it is thus possible that the lower availability of iron in the LowFe group affects the TH regulatory activity.

Significant gene expression changes were not observed for known iron uptake- and storage-related genes among the evaluated conditions. The most probable reasons for these results are: (i) the animals evaluated did not present either anemia or iron overload, but instead extremes from a normal biological distribution; (ii) The skeletal muscle is not the primary target either iron deficiency or overload [59]; (iii) Many genes from the iron metabolism pathways, such as Transferrin receptor 1, Divalent metal transporter 1, Ferroportin, Ferritin L and H, are post-transcriptionally regulated [2,70]. Moreover, Hansen et al. [52] observed that a mild iron deficiency was not enough to cause changes in the expression of intestinal proteins involved in the metabolism of this mineral in beef cattle.

### Candidate genes for beef quality

According to Cánovas et al. [71], meat quality parameters are directly affected by lipid metabolism. Significant correlations between mono- and polyunsaturated fatty acid levels and cholesterol with the iron content in *Semitendinosus* muscle (ST) were observed in crossbred animals [9]. The IMF deposition influences meat juiciness, flavor, texture, and tenderness [49,72]. Accordingly, the *THRSP*, *FASN*, and *FABP4* genes have been suggested as functional candidate genes for intramuscular fat deposition and marbling score in bovine [34,72,73] and swine [71,74]. Although we identified differences in iron content in the gene expression of these candidate genes between iron content groups, they did not appear to affect fat deposition at the time that muscle samples were collected.

The *TNC* and *RCAN1* genes, which were down-regulated in the LowFe group, have been associated with meat quality parameters. In swines, single nucleotide polymorphisms (SNPs) in *TNC* genes were associated with traits such as color, pH, subcutaneous fat, and loin eye area [55]. Also, Jiang et al. [75] identified a pleiotropic effect of the *RCAN1* gene on traits such as carcass weight and beef marbling score.

## Conclusions

Through the transcriptional profile of *Longissimus dorsi* tissue from eight steers genetically divergent for iron content, we identified 49 annotated DE genes. Many of these DE genes are involved in lipogenic functions, such as lipid metabolism, and triglyceride metabolic process. To our knowledge, this is the first study in cattle demonstrating that the variation of iron content in muscle is related to changes in expression variation of lipogenic genes, as well as those related to cell growth and development. This study points to biological routes that are partially influenced by iron, contributing to a better understanding of its participation in pathways that affect beef quality.

## Supporting Information

S1 Fig Transcripts ranked by Cuffmerge. =: Complete correspondence; **i**: A transfrag entirely comprehended inside a reference intron; **o**: exonic generic superposition with one reference transcript; **u**: Unknown, intergenic transcript; **x**: exonic superposition with reference in the opposite strand; **j**: potentially new isoform: at least one splice junction is shared with a reference transcript.
(TIF)

S2 Fig Boxplot of the log_10_ of FPKM values on the conditions of high (HighFe) and low (LowFe) muscle iron content. (TIF)

S3 Fig Hierarchical clustering of the transcriptional profile of the differentially expressed genes between biological replicates with high (HighFe) and low (LowFe) GEBV for muscle iron content. Each line represents a gene, and each column represents an animal. **HIL_0:** High1; **HIL_1:** High2; **HIL_2:** High3; **HIL_3:** High4; **LIL_0:** Low1; **LIL_1:** Low2; **LIL_2:** Low3; **LIL_3:** Low4.
(TIF)

S4 Fig Principal component analysis (PCA) between the conditions of high (HighFe) and low (LowFe) GEBV for muscle iron content. (TIF)

S5 Fig Multi-dimensional scaling between the conditions of high (HighFe) and low (LowFe) GEBV for muscle iron content. **HIL_0:** High1; **HIL_1:** High2; **HIL_2:** High3; **HIL_3:** High4; **LIL_0:** Low1; **LIL_1:** Low2; **LIL_2:** Low3; **LIL_3:** Low4.
(TIF)

S1 Table Mineral content, intramuscular fat content (IMF), and backfat thickness deposition (BFT) in the Nelore *Longissimus dorsi* muscle. (PDF)

S2 Table Functional annotation cluster for differentially expressed genes in the *Longissimus dorsi* muscle, comparing groups with high and low genomic breeding value for iron content in Nelore steers. (PDF)
