# Supplementary figures and images for: Iron Content Affects Lipogenic Gene Expression in the Muscle of Nelore Beef Cattle
Source: PLoS One. 2016 Aug 17;11(8):e0161160. doi: 10.1371/journal.pone.0161160 (PMC4988672; doi:10.1371/journal.pone.0161160)

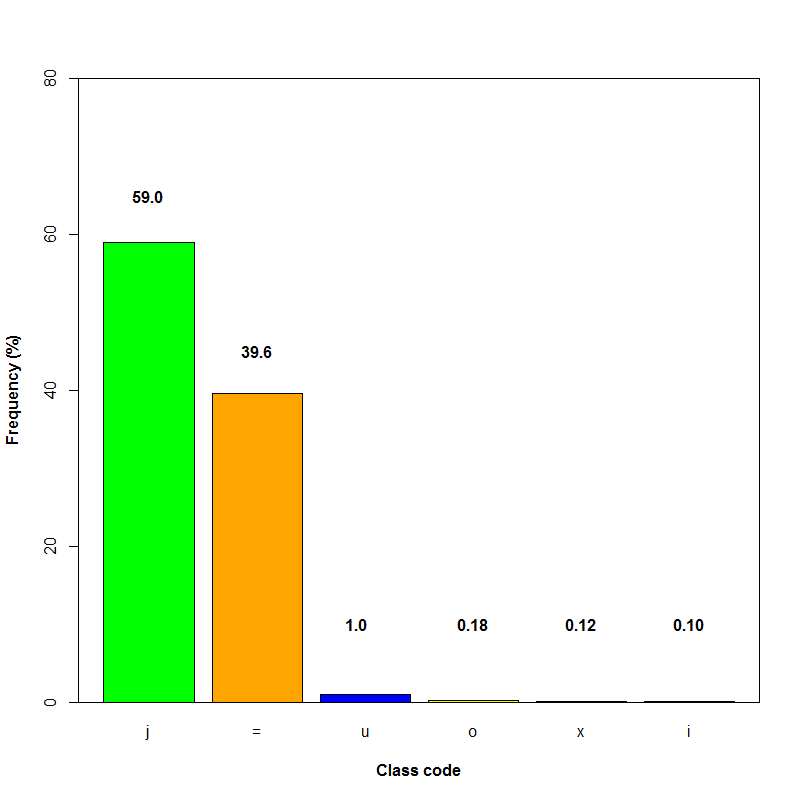

Supplement: S1 Fig — =: Complete correspondence; i: A transfrag entirely comprehended inside a reference intron; o: exonic generic superposition with one reference transcript; u: Unknown, intergenic transcript; x: exonic superposition with reference in the opposite strand; j: potentially new isoform: at least one splice junction is shared with a reference transcript. (TIF) [file pone.0161160.s001.tif]

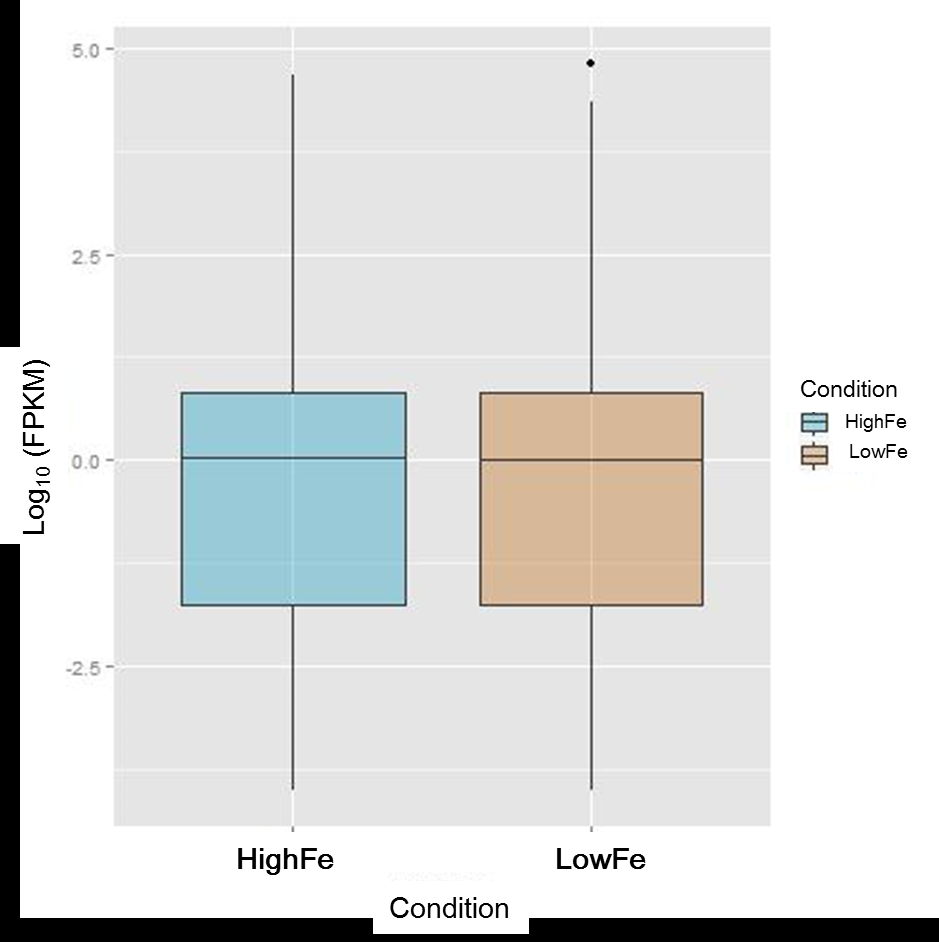

Supplement: S2 Fig — (TIF) [file pone.0161160.s002.tif]

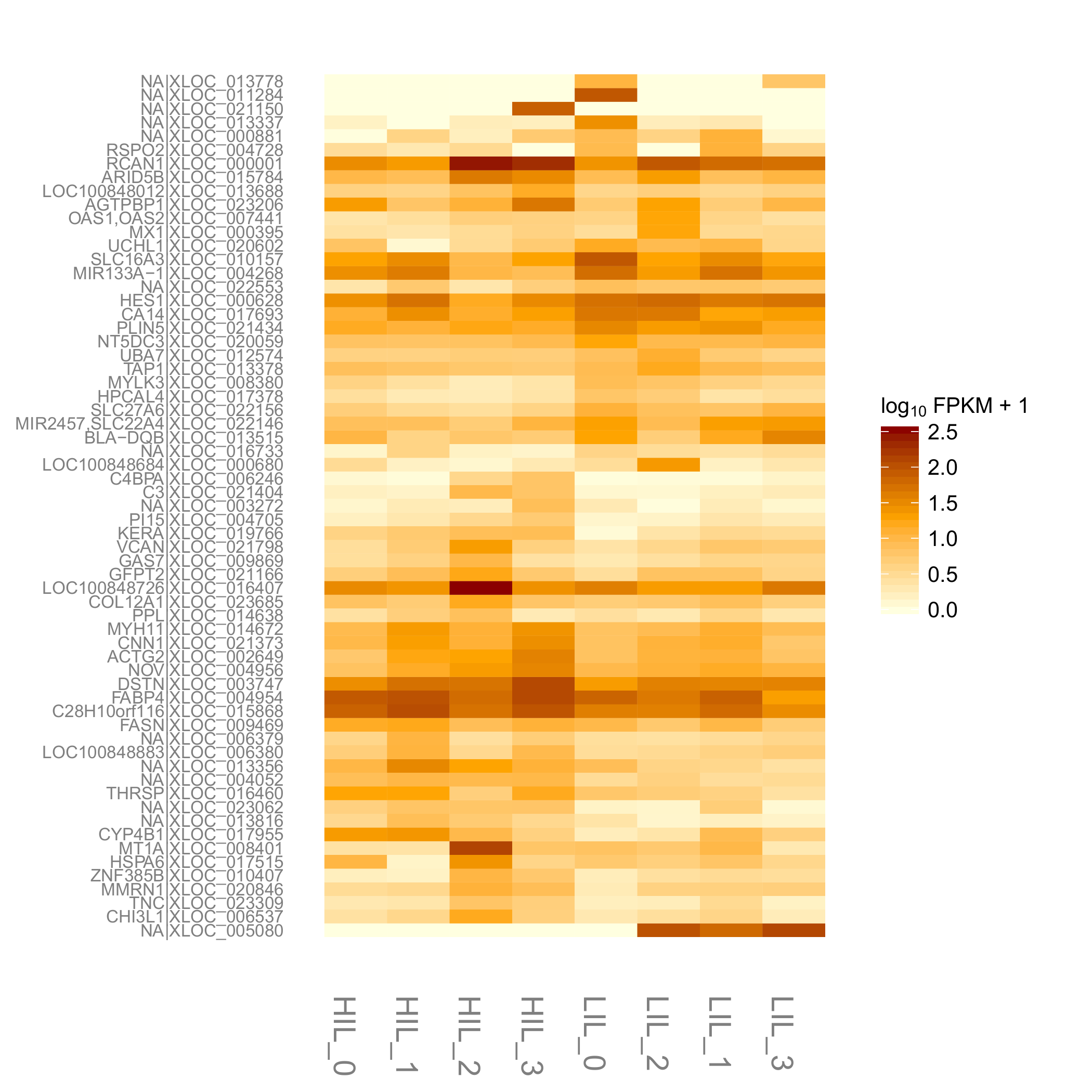

Supplement: S3 Fig — Each line represents a gene, and each column represents an animal. HIL_0: High1; HIL_1: High2; HIL_2: High3; HIL_3: High4; LIL_0: Low1; LIL_1: Low2; LIL_2: Low3; LIL_3: Low4. (TIF) [file pone.0161160.s003.tif]

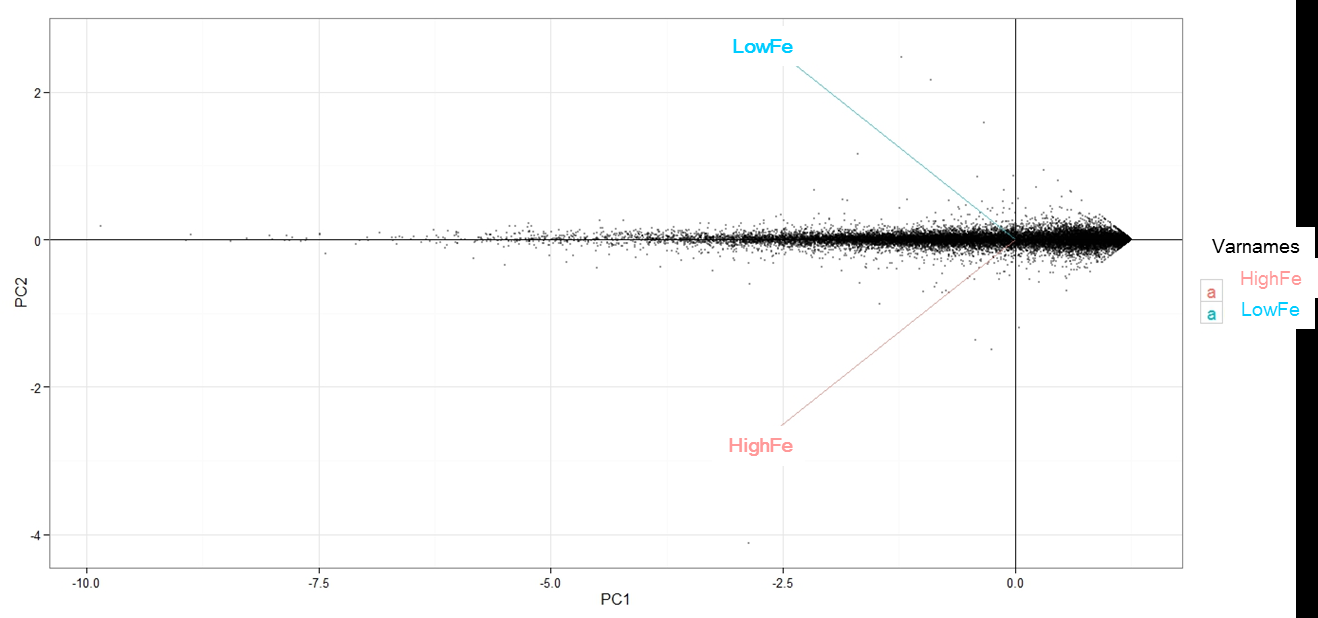

Supplement: S4 Fig — (TIF) [file pone.0161160.s004.tif]

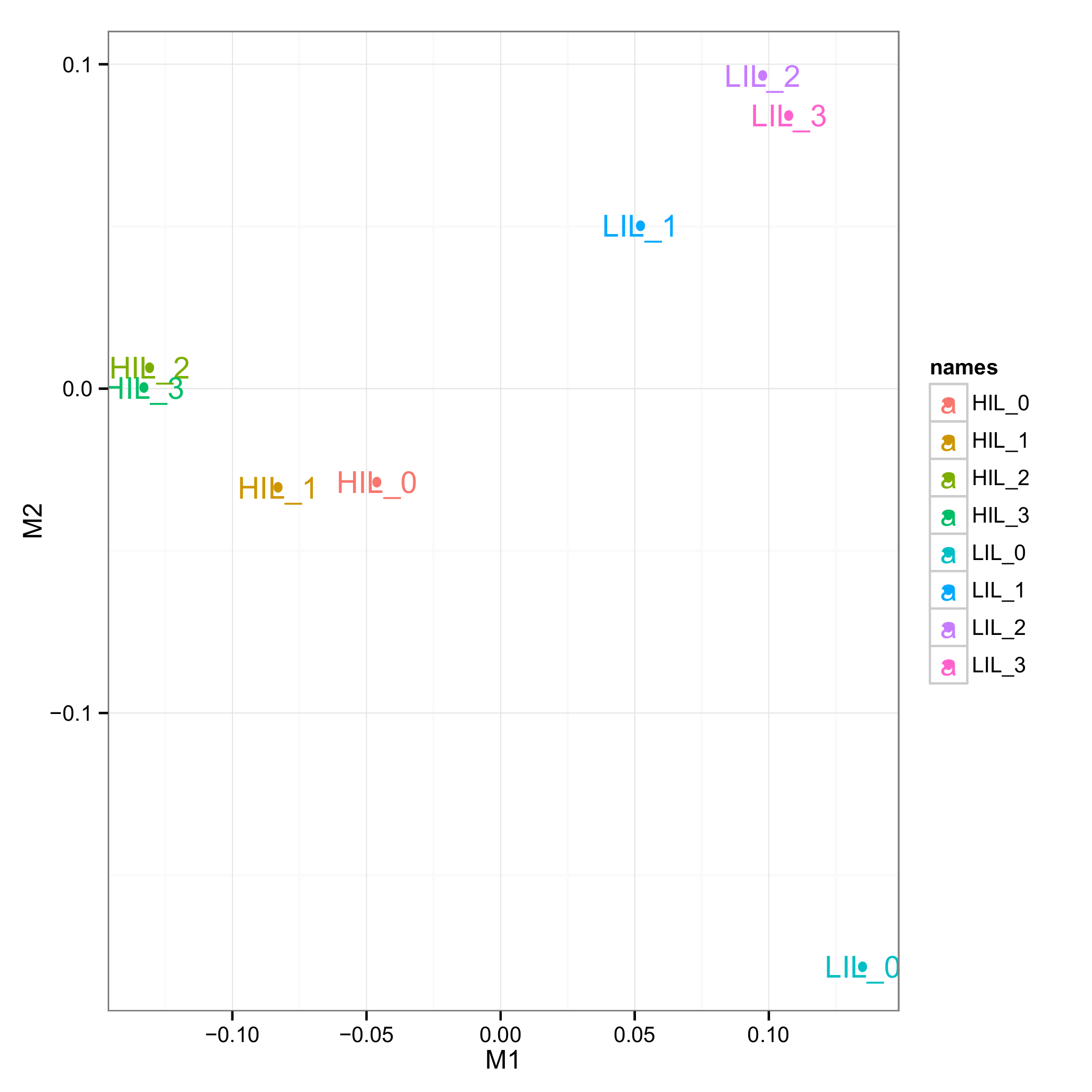

Supplement: S5 Fig — HIL_0: High1; HIL_1: High2; HIL_2: High3; HIL_3: High4; LIL_0: Low1; LIL_1: Low2; LIL_2: Low3; LIL_3: Low4. (TIF) [file pone.0161160.s005.tif]
