# Supplementary material for: Iron Content Affects Lipogenic Gene Expression in the Muscle of Nelore Beef Cattle
Source: PLoS One. 2016 Aug 17;11(8):e0161160. doi: 10.1371/journal.pone.0161160 (PMC4988672; doi:10.1371/journal.pone.0161160)
Supplement: S1 Table — (PDF) [file pone.0161160.s006.pdf]

**Annotation Cluster 1**	**Enrichment Score: 1.8288365043089938**	**PValue**	**Genes**
**Category**	**Term**		
GOTERM_BP_FAT	GO:0048747~muscle fiber development	0.0033	*TNC*, *MYH11*, *RCAN1*
GOTERM_BP_FAT	GO:0055002~striated muscle cell development	0.0071	*TNC*, *MYH11*, *RCAN1*
GOTERM_BP_FAT	GO:0055001~muscle cell development	0.0082	*TNC*, *MYH11*, *RCAN1*
GOTERM_BP_FAT	GO:0007517~muscle organ development	0.0145	*TNC*, *ARID5B*, *MYH11*, *RCAN1*
GOTERM_BP_FAT	GO:0051146~striated muscle cell differentiation	0.0194	*TNC*, *MYH11*, *RCAN1*
GOTERM_BP_FAT	GO:0014706~striated muscle tissue development	0.0339	*TNC*, *MYH11*, *RCAN1*
GOTERM_BP_FAT	GO:0042692~muscle cell differentiation	0.0350	*TNC*, *MYH11*, *RCAN1*
GOTERM_BP_FAT	GO:0060537~muscle tissue development	0.0371	*TNC*, *MYH11*, *RCAN1*
**Annotation Cluster 2**	**Enrichment Score: 1.547720844687776**		
GOTERM_CC_FAT	GO:0031012~extracellular matrix	8.20E-11	*NOV, KERA, TNC, CHI3L1, COL12A1, VCAN*
SP_PIR_KEYWORDS	Secreted	0.0024	*NOV, KERA, C3, TNC, RSPO2, CHI3L1, COL12A1, VCAN, C4BPA, MMRN1, PI15*
GOTERM_CC_FAT	GO:0005578~proteinaceous extracellular matrix	0.0049	*KERA, TNC, CHI3L1, COL12A1, VCAN*
GOTERM_CC_FAT	GO:0005576~extracellular region	0.0078	*NOV, KERA, C3, TNC, RSPO2, CHI3L1, COL12A1, VCAN, C4BPA, MMRN1, PI15*
SP_PIR_KEYWORDS	extracellular matrix	0.0146	*KERA, TNC, COL12A1, VCAN*
GOTERM_CC_FAT	GO:0044421~extracellular region part	0.0159	*NOV, KERA, C3, TNC, CHI3L1, COL12A1, VCAN*
GOTERM_BP_FAT	GO:0009611~response to wounding	0.0388	*C3, TNC, VCAN, C4BPA, MMRN1*
SP_PIR_KEYWORDS	signal	0.0737	*NOV, KERA, CA14, C3, TNC, RSPO2, CHI3L1, COL12A1, VCAN, C4BPA, MMRN1, PI15*
UP_SEQ_FEATURE	signal peptide	0.0765	*NOV, KERA, CA14, C3, TNC, RSPO2, CHI3L1, COL12A1, VCAN, C4BPA, MMRN1, PI15*
SP_PIR_KEYWORDS	disulfide bond	0.0830	*NOV, KERA, CA14, C3, TNC, RSPO2, CHI3L1, COL12A1, VCAN, C4BPA, MMRN1*
GOTERM_BP_FAT	GO:0007155~cell adhesion	0.0888	*HES1, TNC, COL12A1, VCAN, MMRN1*

GOTERM_BP_FAT	GO:0022610~biological adhesion	0.0892	*HES1, TNC, COL12A1, VCAN, MMRN1*
SP_PIR_KEYWORDS	glycoprotein	0.1109	*CA14, KERA, C3, TNC, UCHL1, CHI3L1, C4BPA, MMRN1, NOV, RSPO2, SLC22A4, COL12A1, VCAN, PI15*
UP_SEQ_FEATURE	disulfide bond	0.1405	*NOV, KERA, CA14, C3, TNC, RSPO2, CHI3L1, VCAN, C4BPA, MMRN1*
UP_SEQ_FEATURE	glycosylation site:N-linked (GlcNAc...)	0.1573	*CA14, KERA, C3, TNC, CHI3L1, C4BPA, MMRN1, NOV, RSPO2, SLC22A4, COL12A1, VCAN, PI15*
**Annotation Cluster 3**	**Enrichment Score: 1.1267682736210545**		
GOTERM_BP_FAT	GO:0032989~cellular component morphogenesis	0.0153	*HES1, UCHL1, MYH11, VCAN, GAS7*
GOTERM_BP_FAT	GO:0030182~neuron differentiation	0.0211	*HES1, AGTPBP1, UCHL1, VCAN, GAS7*
GOTERM_BP_FAT	GO:0048666~neuron development	0.0489	*HES1, UCHL1, VCAN, GAS7*
GOTERM_BP_FAT	GO:0000902~cell morphogenesis	0.0551	*HES1, UCHL1, VCAN, GAS7*
GOTERM_BP_FAT	GO:0048667~cell morphogenesis involved in neuron differentiation	0.0918	*HES1, UCHL1, VCAN*
GOTERM_BP_FAT	GO:0048812~neuron projection morphogenesis	0.0948	*UCHL1, VCAN, GAS7*
GOTERM_BP_FAT	GO:0000904~cell morphogenesis involved in differentiation	0.1189	*HES1, UCHL1, VCAN*
GOTERM_BP_FAT	GO:0048858~cell projection morphogenesis	0.1197	*UCHL1, VCAN, GAS7*
GOTERM_BP_FAT	GO:0031175~neuron projection development	0.1286	*UCHL1, VCAN, GAS7*
GOTERM_BP_FAT	GO:0032990~cell part morphogenesis	0.1286	*UCHL1, VCAN, GAS7*
GOTERM_BP_FAT	GO:0030030~cell projection organization	0.2261	*UCHL1, VCAN, GAS7*
**Annotation Cluster 4**	**Enrichment Score: 1.1030523645221304**		
GOTERM_BP_FAT	GO:0009611~response to wounding	0.0388	*C3, TNC, VCAN, C4BPA, MMRN1*
INTERPRO	IPR006209:EGF	0.0390	*TNC, VCAN, MMRN1*
INTERPRO	IPR000742:EGF-like, type 3	0.0823	*TNC, VCAN, MMRN1*
SP_PIR_KEYWORDS	egf-like domain	0.0862	*TNC, VCAN, MMRN1*
INTERPRO	IPR006210:EGF-like	0.0875	*TNC, VCAN, MMRN1*
GOTERM_BP_FAT	GO:0007155~cell adhesion	0.0888	*HES1, TNC, COL12A1, VCAN, MMRN1*

GOTERM_BP_FAT	GO:0022610~biological adhesion	0.0892	*HES1, TNC, COL12A1, VCAN, MMRN1*
SMART	SM00181:EGF	0.0980	*TNC, VCAN, MMRN1*
INTERPRO	IPR013032:EGF-like region, conserved site	0.1622	*TNC, VCAN, MMRN1*
**Annotation Cluster 5**	**Enrichment Score: 1.0757278267449475**		
SP_PIR_KEYWORDS	nucleotide-binding	0.0080	*ACTG2, MYLK3, TAP1, MYH11, SLC22A4, UBA7, HSPA6, SLC27A6, OAS1, MX1*
SP_PIR_KEYWORDS	atp-binding	0.0208	*ACTG2, MYLK3, TAP1, MYH11, SLC22A4, UBA7, HSPA6, OAS1*
GOTERM_MF_FAT	GO:0005524~ATP binding	0.0957	*ACTG2, MYLK3, TAP1, MYH11, SLC22A4, UBA7, HSPA6, OAS1*
GOTERM_MF_FAT	GO:0032559~adenyl ribonucleotide binding	0.1011	*ACTG2, MYLK3, TAP1, MYH11, SLC22A4, UBA7, HSPA6, OAS1*
GOTERM_MF_FAT	GO:0032553~ribonucleotide binding	0.1114	*ACTG2, MYLK3, TAP1, MYH11, SLC22A4, UBA7, HSPA6, OAS1, MX1*
GOTERM_MF_FAT	GO:0032555~purine ribonucleotide binding	0.1114	*ACTG2, MYLK3, TAP1, MYH11, SLC22A4, UBA7, HSPA6, OAS1, MX1*
GOTERM_MF_FAT	GO:0030554~adenyl nucleotide binding	0.1246	*ACTG2, MYLK3, TAP1, MYH11, SLC22A4, UBA7, HSPA6, OAS1*
GOTERM_MF_FAT	GO:0001883~purine nucleoside binding	0.1321	*ACTG2, MYLK3, TAP1, MYH11, SLC22A4, UBA7, HSPA6, OAS1*
GOTERM_MF_FAT	GO:0017076~purine nucleotide binding	0.1346	*ACTG2, MYLK3, TAP1, MYH11, SLC22A4, UBA7, HSPA6, OAS1, MX1*
GOTERM_MF_FAT	GO:0001882~nucleoside binding	0.1357	*ACTG2, MYLK3, TAP1, MYH11, SLC22A4, UBA7, HSPA6, OAS1*
GOTERM_MF_FAT	GO:0000166~nucleotide binding	0.1377	*ACTG2, MYLK3, TAP1, MYH11, SLC22A4, UBA7, HSPA6, SLC27A6, OAS1, MX1*
UP_SEQ_FEATURE	nucleotide phosphate-binding region:ATP	0.1497	*MYLK3, TAP1, MYH11, SLC22A4, UBA7*
**Annotation Cluster 6**	**Enrichment Score: 0.8586899310100715**		
GOTERM_BP_FAT	GO:0030036~actin cytoskeleton organization	0.0174	*MYH11, CNN1, GAS7, DSTN*
GOTERM_CC_FAT	GO:0015629~actin cytoskeleton	0.0205	*ACTG2, MYH11, GAS7, DSTN*
GOTERM_BP_FAT	GO:0030029~actin filament-based process	0.0206	*MYH11, CNN1, GAS7, DSTN*
**Annotation Cluster 7**	**Enrichment Score: 0.8124139950661177**		
SP_PIR_KEYWORDS	immune response	0.0824	*C3, TAP1, C4BPA*
