# Supplementary material for: Iron Content Affects Lipogenic Gene Expression in the Muscle of Nelore Beef Cattle
Source: PLoS One. 2016 Aug 17;11(8):e0161160. doi: 10.1371/journal.pone.0161160 (PMC4988672; doi:10.1371/journal.pone.0161160)
Supplement: S2 Table — (PDF) [file pone.0161160.s007.pdf]

Animal	Category	Minerals (mg/kg)			IMF (%)	BFT (mm)
		**Cu**	**Mn**	**Zn**		
Low1	LowFe*	1,53	0,093	106,48	3,75	6,00
Low2	LowFe	2,25	0,194	106,23	3,99	15,00
Low3	LowFe	1,41	0,100	93,21	1,58	9,00
Low4	LowFe	1,54	0,021	128,23	2,20	4,00
**Mean**		**1,68** a	**0,10** a	**108,54** a	**2,88** a	**8,50** a
High1	HighFe**	1,51	0,250	99,44	3,61	8,00
High2	HighFe	1,14	0,016	109,68	2,84	2,50
High3	HighFe	1,22	0,009	99,75	1,39	4,00
High4	HighFe	1,29	0,014	113,74	1,38	6,00
**Mean**		**1,29** a	**0,07** a	**105,65** a	**2,31** a	**5,13** a

*LowFe – low iron content group; **HighFe – high iron content group. Means with the same letter in the column are not significantly different from each other, by t-test ($P > 0.05$).
